# Characterization of Avian Pathogenic *Escherichia coli* (APEC) Associated With Turkey Cellulitis in Iowa

**DOI:** 10.3389/fvets.2020.00380

**Published:** 2020-07-03

**Authors:** Aline Luisa de Oliveira, Darby M. Newman, Yuko Sato, Andrew Noel, Britney Rauk, Lisa K. Nolan, Nicolle L. Barbieri, Catherine M. Logue

**Affiliations:** ^1^Department of Population Health, College of Veterinary Medicine, University of Georgia, Athens, GA, United States; ^2^Department of Veterinary Diagnostic and Production Animal Medicine, College of Veterinary Medicine, Ames, IA, United States; ^3^Department of Infectious Diseases, College of Veterinary Medicine, University of Georgia, Athens, GA, United States

**Keywords:** *Escherichia coli*, APEC, Turkey cellulitis, characterization, poultry

## Abstract

Turkey cellulitis, also known as clostridial dermatitis is a significant cause of morbidity, mortality, and carcass condemnation at slaughter resulting in considerable losses for turkey producers. Here, we assessed the potential role of Avian Pathogenic *Escherichia coli* (APEC) in a cellulitis outbreak on a turkey farm in Iowa. Birds from one farm with a history of cellulitis and one farm with no history of disease (for comparison) were followed from the age of 10 weeks (before the outbreak) to 18 weeks (just prior to slaughter). *E. coli* recovered from the litter, from skin lesions of birds with cellulitis, and from systemic lesions of birds submitted for necropsy, were assessed. A total of 333 isolates were analyzed and screened for virulence-associated genes, antimicrobial resistance genes including heavy metal resistance, adhesins, invasins, and protectins, iron acquisition systems and their phylogenetic group through multiplex PCR. In addition, PCR was used to serogroup the isolates, and pulsed field gel electrophoresis (PFGE) was used to analyze a subset of strains from the farm environment (litter) and birds at 17 and 18 weeks of age when the cellulitis infection appeared to peak. Overall, *E. coli* isolates recovered from cellulitis lesions and systemic infection were identified as APEC, while a lower prevalence of *E. coli* recovered from the litter met the criteria of APEC-like. Direct comparison of *E. coli* isolates from the litter, lesions, and systemic strains using PFGE failed to find identical clones across all three sources reflecting the diversity of strains present in the poultry environment causing disease. This study highlights the role of APEC in turkey cellulitis and should not be overlooked as a significant contributor to the disease in turkeys.

## Introduction

The United States produced 245.2 million turkeys in 2017 and an estimated 244.75 million for 2018 (1), resulting in an industry that generates 4–4.5 billion dollars in value. The State of Iowa occupies seventh position in production ranks with 11.9 million birds produced in 2018 representing 6.8% of the total national production (USDA) (1).

Colibacillosis is considered one of the leading bacterial causes of economic loss in the turkey industry worldwide (2). Turkey cellulitis (TC) is also among the top health issues in turkey producers nationally, ranking as #3 in 2019 according to the United States Animal Health Association turkey industry survey (3). Avian pathogenic *Escherichia coli* (APEC) is one of the pathogens implicated in the disease after *Clostridium* spp. Cellulitis (also known as clostridial dermatitis) is characterized by locally extensive inflammation of subcutaneous tissues of the inguinal, tail, and/or breast regions, often striking production toms at or near market age resulting in increased mortality and carcass condemnation at slaughter, leading to multi-million dollar losses for the turkey industry (4–6). Information on the role of *E. coli* in cellulitis-associated disease in turkeys is relatively limited as most often clostridia species in particular *Cl. septicum, Cl. perfringens, Cl. sordelli* have been implicated as the etiologic agents (5, 7). In addition, other microorganisms including *Streptococcus* spp. and *Staphylococcus aureus* have also been identified as potential agents of disease (5, 8, 9). Of significant interest to the current study is the view that *E. coli* is an infrequent cause of cellulitis in turkeys (9). Damage to tissues as a result of cellulitis often have their origin as a result of a trauma or injury to the bird and studies have demonstrated the importance of quality litter that is less likely to cause trauma as well as the microbiome of the litter which has the potential to affect skin health in injured or compromised birds. Also of importance is the potential for the environment to harbor clostridia spores, which as a pathogen can contribute to the tissue destruction resulting in similar tissue damage (5). In an effort to better understand the role of *E. coli* in cellulitis-associated disease in turkeys we assessed the quality of litter, lesions from birds and systemic isolates recovered from infected birds to assess the potential role of APEC in turkey cellulitis.

## Materials and Methods

### Sample Collection—Farms

Weekly litter samples and swabs of cellulitis lesions and birds with cellulitis were collected during the course of this study. Convenience sampling was conducted based on producer observation of infection in birds rather than sample size calculation since infection could not be predicted. Three barns were visited on a weekly basis for sample collection. Two of the barns had no history of cellulitis-associated disease (control barns) in the past 12 months (Barns A1 and B1), and the third barn had a cellulitis outbreak (case barn, Barn A2). A case farm was defined as a farm in which at least 2/3 of the flocks placed were affected with cellulitis during the previous 12 months, and an outbreak was defined when mortality due to cellulitis exceeds 0.5 per 1,000 birds for 2 consecutive days. Barn A1 was visited on eight separate occasions, starting when the turkeys were 10 weeks of age, and ending when they were 17 weeks old (pre-slaughter). Barn A2 was visited seven times, staring when the turkeys were 12 weeks old, and ending when they were 18 weeks (pre-slaughter). Barn B1 was visited six times, beginning at 12 weeks of age and ending at 17 weeks old (pre-slaughter). Upon arrival at each respective facility, weekly mortality and any antimicrobial treatments were noted. Each barn was divided into four quadrants, divided by fans or other markers present in the facility. A trowel was used to gather a sample of litter ~7 cm in diameter and 4 cm deep from a random location in each quadrant. The litter was collected in a sterile whirlpak bag (Whirlpak, Nasco, Fort Atkinson, WI) and placed in an ice chest containing ice packs for transport to the lab for analysis. Four samples of litter (one per quadrant) were collected on each visit date. Common litter components included pine shavings and oat hulls mixed with sand, spread over a base of packed clay or dirt.

### Tissue Sampling/Collection

To collect samples of cellulitis lesions, we relied on producer-diagnosed cases of cellulitis in birds found dead upon walking through the barn. The only barn with an outbreak of cellulitis in this study was barn A2, as defined above in sample collection. When mortality was present in cellulitis barns, up to 5 birds per week were harvested, and necropsies were performed in the field and at Iowa State University's Veterinary Diagnostic Laboratory (ISU-VDL). From a total of 11 birds, 35 lesion samples were taken using sterile cotton swabs. Swabs of the subcutis, where cellulitis lesions occur, were taken from areas of crepitus and blistering, usually on the breast, and from areas of fluid accumulation, edema, and inflammation, typically near the thigh and ventral to the inguinal region. Before any incisions were made into the affected tissue, the area was flame sterilized to avoid cross contamination. Then, using flame sterilized instruments, the skin was incised to gain access to the subcutis, a swab was inserted, and the area swabbed to include the affected tissue, the swab was then placed in a sterile Cary Blair transport media (BBL, Becton Dickinson, Franklin, NJ) tube for processing in the diagnostic microbiology lab.

### Sample Processing

To process the litter sample, the procedure described by Lu et al. (10) was adapted to fit the facilities and testing procedures available for this study. Each quadrant sample was processed individually, allowing for a better profile of the barn than pooling the samples. The quadrant litter sample was mixed thoroughly, and a 2.5 g sub-sample was placed into a clean glass tube containing 15 mL of phosphate buffered saline (litter wash) (PBS, Research Products International Corp., Mt Prospect, IL). The sample was vortex mixed on the highest setting for ~5–10 s, and allowed to stand briefly before pipetting 2 mL of solution into each of two, 2 mL centrifuge tubes, one for analysis and one for preservation. The tubes were centrifuged at 100 × *g* for 5 min to pellet large debris. The supernatant was decanted into a new tube and centrifuged at 12,000 × *g* for 5 min to pellet all bacteria in the solution. The supernatant from that tube was discarded, and the remaining pellet was re-suspended in 1 mL of PBS. Ten microliter of that solution was streaked onto a MacConkey agar plate using an inoculating loop and incubated overnight at 37°C. In addition, the remaining litter wash (13 ml) was incubated for 18 h at 37°C and struck (10 μL) to MacConkey agar as a means to pick up additional samples that may not have been positive on initial testing from, the litter wash directly.

To process the lesion sample, 2 mL of PBS was added to each tube containing a swab. The tube was closed and mixed by vortex at the highest setting. A 10 μL portion of the solution was spread on one half of a MacConkey agar plate using an inoculating loop, and the same swab was touched to the other half of the plate and spread evenly using the same inoculating loop. The plate was then incubated at 37°C overnight.

### Tissue Analysis

Sections of liver, spleen, skin, and muscle tissue were submitted to ISU's VDL for microbial analysis. From those submissions, a total of 39 *E. coli* isolates were recovered, with identity confirmed by MALDI-TOF (Bruker, Billerica, MA). They were re-struck from pure cultures onto MacConkey plates and incubated overnight at 37°C for further analysis as described below.

### *E. coli* Analysis

After the plates of each sample type had grown, colonies were selected for testing. From each litter sample, three colonies were selected, totaling 12 isolates per barn per week. From each affected bird, a total of five colonies were selected from all of the swabs taken from that bird. Since the goal of this study was to identify APEC in both sample types, after overnight growth, colonies were selected for testing based on qualitative characteristics commonly found on known *E. coli* colonies. Lactose positive, dimpled, and circular colonies were chosen over lactose negative, irregularly shaped colonies. When there were not three colonies fitting that description, lactose positive colonies representative of the growth on the plate were selected.

A total of 333 *E. coli* isolates (240 litter, 54 cellulitis, and 39 systemic), were isolated from all samples collected and analyzed with regard to virulence-associated genes profile (including antimicrobial resistance genes, heavy metal resistance genes, cell surface structures, iron acquisition systems), phylogenetic group, and serogroup through multiplex PCR.

### DNA Extraction

Bacterial DNA was obtained from whole organisms using the boil prep method. Briefly, isolates were grown at 37°C overnight on LB agar. Next, an isolated colony was inoculated into 1 ml of LB broth and grown overnight at 37°C. Cultures were centrifuged at 12,000 rpm for 3 min, the supernatant was discarded and the cells were re-suspended in 200 μL of molecular-grade water and boiled 100°C for 10 min, allowed to cool and centrifuged at 12,000 rpm for 3 min to precipitate cellular debris; 150 μL of the supernatant was transferred to a new tube and used as DNA template for gene amplification. The DNA extracts were stored at −20°C until use.

### *E. coli* Confirmation

To confirm the selected colonies were *E. coli*, isolates were screened by PCR of the 16S DNA as described previously by Lamprecht et al. (11). Amplification was carried out in an Eppendorf Mastercycler (Eppendorf, Hamburg, Germany) with the following parameters: 94°C for 3 min; 35 cycles of 94°C for 30 s, 60°C for 30 s, 72°C for 1 min; followed by a final extension of 72°C for 10 min. PCR products were subjected to horizontal gel electrophoresis in a 2% agarose gel (LE Agarose, Lonza, Alpharetta, GA) at 100 V for 70 min. A Hi-Lo molecular weight marker (100 bp; Minnesota Molecular, Minneapolis, MN) and negative (sterile water) and positive controls from our lab collections were included on the gel for comparison and confirmation purposes. After electrophoresis, the gel was stained in 0.25% Ethidium Bromide solution (Sigma Aldrich. St. Louis, MO) for 20 min and viewed under UV light using an Omega Lum G imager (Aplegen, San Francisco, CA). Isolates were recorded as positive or negative for the 16S.

### APEC Minimal Predictors

Screening of *E. coli* for detection of genes that define the APEC pathotype was performed by genotyping the isolates for *iroN, ompT, hlyF, iss*, and *iutA*, defined as minimal APEC predictors by Johnson et al. (12). Isolates with three or more of these genes were classified as APEC if isolated from a lesion of disease (13) or APEC-like when recovered from non-disease samples (litter, feces etc.). Additional analysis included screening for 64 virulence and antimicrobial resistance-associated genes, phylogenetic analysis and PCR-based O-typing of 240 litter, 54 cellulitis and 39 systemic isolates.

### Multiplex PCRs

The presence of genes encoding virulence factors was investigated through multiplex polymerase chain reaction (PCR) amplification as previously described (14, 15). Nine multiplex PCR panels were developed to detect 64 virulence genes. Primer sequences and gene definitions are shown in Table 1. Reactions were performed in 25 μl volume containing 2.5 μl of 10x PCR buffer, 0.4 μl 50 mM MgCl_2_, 1.25 μl dNTP (10 μM) Pool, 2 U Taq DNA polymerase, 0.075 μl (200 μM) of each primer and 2 μl of DNA sample. The conditions for the reactions were as follows, except for the annealing temperature that was adjusted according to the multiplex: 94°C for 5 min; 30 cycles of 94°C for 30 s, 63°C for 30 s, 68°C for 10 min, and a final extension step of 72°C for 10 min. Annealing temperatures were as follows: 63°C for multiplex 1, 2, 3, 4, 5, and 17; 60°C for multiplex 6 and 12; and 58°C for multiplex 8.

**Table 1:** Primers sets and multiplex PCRs used in analysis of APEC.

**Gene**	**Amplicon size (bp)**	**Primer sequence (5′-3′)**	**Description**	**References**
**MULTIPLEX 1**				
*malX*	925	GGACATCCTGTTACAGCGCGCA		
		TCGCCACCAATCACAGCCGAAC		
*papA*	717	ATGGCAGTGGTGTTTTGGTG	*Pap* operon	(16)
		CGTCCCACCATACGTGCTCTTC		
*fimH*	508	TCGAGAACGGATAAGCCGTGG	Type 1 fimbriae	(16)
		GCAGTCACCTGCCCTCCGGTA		
*kpsIII*	392	TCCTCTTGCTACTATTCCCCCT	Capsule	(16)
		AGGCGTATCCATCCCTCCTAAC		
papEF	326	GCAACAGCAACGCTGGTTGCATCAT	*Pap* operon	(16)
		AGAGAGAGCCACTCTTATACGGACA		
*ireA*	254	GATGACTCAGCCACGGGTAA	Iron acquisition	(15)
		CCAGGACTCACCTCACGAAT		
*ibeA*	171	AGGCAGGTGTGCGCCGCGTAC	Invasin	(16)
		TGGTGCTCCGGCAAACCATGC		
**MULTIPLEX 2**				
*cnf-1*	1,105	ATCTTATACTGGATGGGATCATCTTGG	Cytotoxic necrotizing factor	(16)
		GCAGAACGACGTTCTTCATAAGTATC		
*fyuA*	787	TGATTAACCCCGCGACGGGAA	Iron acquisition	(16)
		CGCAGTAGGCACGATGTTGTA		
*ironEC*	667	AAGTCAAAGCAGGGGTTGCCCG	Salmochelin	(15)
		GACGCCGACATTAAGACGCAG		
*bmaE*	507	ATGGCGCTAACTTGCCATGCTG	Heme-agglutinin	(16)
		AGGGGGACATATAGCCCCCTTC		
*sfa-foc*	410	CTCCGGAGAACTGGGTGCATCTTAC	Fimbriae	(16)
		CGGAGGAGTAATTACAAACCTGGCA		
*aerJ*	302	GGCTGGACATCATGGGAACTGG	Aerobactin	(16)
		CGTCGGGAACGGGTAGAATCG		
*papGIII*	258	GGCCTGCAATGGATTTACCTGG	*Pap* operon	(16)
		CCACCAAATGACCATGCCAGAC		
**MULTIPLEX 3**				
*hlyD*	904	CTCCGGTACGTGAAAAGGAC	Hemolysin D	(15)
		GCCCTGATTACTGAAGCCTG		
*rfc*	788	ATCCATCAGGAGGGGACTGGA	O antigen polymerase	
		AACCATACCAACCAATGCGAG		
*ompT*	559	ATCTAGCCGAAGAAGGAGGC	OM protein	(15)
		CCCGGGTCATAGTGTTCATC		
*papGI'*	479	CTACTATAGTTCATGCTCAGGTC	*Pap* operon	(16)
		CCTGCATCCTCCACCATTATCGA		
*papGI*	461	TCGTGCTCAGGTCCGGAATTT	*Pap* operon	(16)
		TGGCATCCCCCAACATTATCG		
*kpsII*	272	GCGCATTTGCTGATACTGTTG	Capsule	(16)
		CATCCAGACGATAAGCATGAGCA		
*papC*	205	GTGGCAGTATGAGTAATGACCGTTA	*Pap* operon	(16)
		ATATCCTTTCTGCAGGGATGCAATA		
**MULTIPLEX 4**				
*gafD*	952	TGTTGGACCGTCTCAGGGCTC	Fimbria	(16)
		TCCCGGAACTCGCTGTTACT		
*cvaC*	679	CACACACAAACGGGAGCTGTT	*colIV* operon	(16)
		CTTCCCGCAGCATAGTTCCAT		
*fliC*	547	ACGATGCAGGCAACTTGACG	Flagellar gene	(15)
		GGGTTGGTCGTTGCAGAACC		
*cdtS*	430	GAAAATAAATGGAACACACATGTCCG	Toxin	(16)
		GAAAGTAAATGGAATATAAATGTCCG		
*focG*	364	CAGCACAGGCAGTGGATACGA	Fimbrial subunit	(16)
		GAATGTCGCCTGCCCATTGCT		
*traT*	290	GGTGTGGTGCGATGAGCACAG	Complement resistance	(16)
		CACGGTTCAGCCATCCCTGAG		
*papGII*	190	GGGATGAGCGGGCCTTTGAT	*Pap* operon	(16)
		CGGGCCCCCAAGTAACTCG		
**MULTIPLEX 5**				
*papGI*	1,140	CTGTAATTACGGAAGTGATTTCTG	*Pap* operon	
		TTCCAGAAATAGCTCATGTAACCCG		
*papGII&III*	1,070	CTGTAATTACGGAAGTGATTTCTG	*Pap* operon	
		ACTATCCGGCTCCGGATAAACCAT		
*iha*	829	CTGGCGGAGGCTCTGAGATCA	UPEC island	
		TCCTTAAGCTCCCGCGGCTGA		
*afa*	594	GGCAGAGGGCCGGCAACAGGC	Adhesin Afa	
		CCCGTAACGCGCCAGCATCTC		
*iss*	323	CAGCAACCCGAACCACTTGATG	Increased serum survival	(17)
		AGCATTGCCAGAGCGGCAGAA		
*sfaS*	244	GTGGATACGACGATTACTGTG	Fimbrial minor subunit	(16)
		CCGCCAGCATTCCCTGTATTC		
*K1*	153	TAGCAAACGTTCTATTGGTGC	Capsule	(16)
		CATCCAGACGATAAGCATGAGCA		
**MULTIPLEX 6**				
*hlyF*	599	GGCGATTTAGGCATTCCGATACTC	Hemolysin F	
		ACGGGGTCGCTAGTTAAGGAG		
*etsB*	537	CAGCAGCGCTTCGGACAAAATCTCCT	*E. coli* transport system	
		TTCCCCACCACTCTCCGTTCTCAAAC		
*colM*	498	CAGCGCCATTACCCCATAAATAGTGA	Colicin M	
		GGTTCGTTCGCCGGTGTAAGCGTTAG		
*etsA*	450	CAACTGGGCGGGAACGAAATCAGGA	*E. coli* transport system	
		TCAGTTCCGCGCTGGCAACAACCTAC		
*colB*	430	ACAAGACAGCACCAGTTATGGGTATT	Colicin B	
		GTTGTTGGTTTTGTTGGCGTAGTTAT		
*eitB*	380	TGATGCCCCGCCAAACTCAAGA	Iron transport system	
		ATGCGCCGGCCTGACATAAGTGCTAA		
*eitA*	284	ACGCCGGGTTAATAGTTGGGAGATAG	Iron transport system	
		ATCGATAGCGTCAGCCCGGAAGTTAG		
**MULTIPLEX 8**				
*blaTEM*	558	ATGTGCGCGGAACCCCTATTTGTTTA	Ampicillin resistance	
		AAAAAGCGGTTAGCTCCTTCGGTCCT		
*aac3VI*	502	GGCACCCGCGACGCCCTGGTCCAAAAG	Gentamicin resistance	
		GGGCCCGGCGCCGATCGACAGGATTT		
*tetB*	446	AACGCGTGAAGTGGTTCGGTTGGT	Tetracycline resistance	
		TTCGCCCCATTTAGTGGCTATTCTTC		
*tetA*	372	CGGGGCGACTGGGGCGGTAGC	Tetracycline resistance	
		CAAAGCGCGGCCGGCACCTGT		
*groEL*	318	CGCCGGCATGAACCCGATGGACCTCA	Chaperone	
		TCGGCCTGCATCGACTGCGGGTTGTTG		
*aph(3)IA*	278	TCGGGCAATCAGGTGCGACAATCTA	Gentamicin resistance	
		TGCCAGCGCATCAACAATATTTTCACC		
*drf17*	243	ATATCCCGTGGTCAGTAAAAGGTG	Trimethoprim resistance	
		GACCCCCGCCAGAGACATA		
**MULTIPLEX 12**				
*terX*	576	ATGCGCCGCCTGCCTGTTTACCTTGTTA	Tellurite resistance	
		CGCGCTTGTGCTGCCGGAAGACA		
*pcoA*	507	ATCCGGAAGGTCAGCACCGTCCATAGAC	Copper resistance	
		GACCTCGCGGATGTCAGTGGCTACACCT		
*terF*	428	CCGACAAACTTCCAGAAGATGGGGTAGT	Tellurite resistance	
		GAGGCAGCGGTTGCATTTGTACTTGACG		
*pcoE*	385	GTGGGGCAGCTTTTGCTCAGTCCAGTGA	Copper resistance	
		CGAAGCTTTCTTGCCTGCGTCTGATGTG		
*terY3*	302	CCTGGGGCCGTCAGCGGACCTG	Tellurite resistance	
		TCCTTGCTGGTGGCCGTTCATACTTCAT		
*terD*	231	CCACTGCGCGGAATTTCCACTCACCAT	Tellurite resistance	
		ACGCCGTCCCGTCTGATGTTGACAAG		
*arsC*	153	CCAGCCTGCGGCACCTCGCGTAATAC	Arsenic resistance	
		ACGCAGCAGCGCTCGTACTGAAATACCC		
**MULTIPLEX 17**				
*silP*	603	ACACCCCGGCCTGGGCTCCTT	Silver resistance	
		TGCGGGCACGGGAACAAACCTC		
*intl1*	545	CACTCCGGCACCGCCAACTTTC	Integrase	
		GAACGGGCATGCGGATCAGTGAG		
*pcoD*	502	GGCGCCCAGAATGATAATCGCAACA	Copper resistance	
		GGGCGTGGCGCTGGCTACACTT		
*sulI*	462	CGCCGCTCTTAGACGCCCTGTCC	Sulfa resistance	
		CAACGGTGGCGCCCAAGAAGGAT		
*iseC12*	404	CGCGGCCACGTAAACCGAAAGATAAA	Transposase	
		GCGCGGGTGCACAGCAACCTC		
*aadA*	365	TAACGGCGCAGTGGCGGTTTTCA	Aminoglycoside resistance	
		AAGCTCGCCGCGTTGTTTCATCAAG		
*aac3VI*	302	GGGCAAGCGCCGCGTCACTTATT	Gentamicin resistance	
		CGCGGCGTTGTTTCGGCTTCA		
*qacEdelta1*	246	TCGGCCTCCGCAGCGACTTCC	Ammonium resistance	
		CTTGCCCCTTCCGCCGTTGTCTAAT		
**PHYLOGENETIC TYPING**				
*chuA*	288	ATGGTACCGGACGAACCAAC		(18)
		TGCCGCCAGTACCAAAGACA		(19)
*yjaA*	211	CAAACGTGAAGTGTCAGGAG		(18)
		AATGCGTTCCTCAACCTGTG	Quadruplex PCR	(18)
TspE4.C2	152	CACTATTCGTAAGGTCATCC		(18)
		AGTTTATCGCTGCGGGTCGC		(18)
*arpA*	400	AACGCTATTCGCCAGCTTGC		(18)
		TCTCCCCATACCGTACGCTA		(20)
*arpA1*	301	GATTCCATCTTGTCAAAATATGCC	Group E	(21)
		GAAAAGAAAAAGAATTCCCAAGAG		(21)
*trpA*	219	AGTTTTATGCCCAGTGCGAG	Group C	(21)
		TCTGCGCCGGTCACGCCC		(21)

### PCR-Based Phylogenetic Classification

The phylogenetic group of the isolates was determined according to the *E. coli* phylogenetic typing method described by Clermont et al. (18), which assigns *E. coli* strains to the phylogenetic groups A, B1, B2, C, D, E, and F. First, a quadruplex PCR was performed for the genes *chuA, yjaA*, and *arpA*, and the DNA fragment *TSPE4.C2*. Depending on the band pattern of the isolate, it was classified as A or C, D or E, B1, C, F and B2 or *E* clades. If an isolate showed a band pattern that could classify it as both A or C, or both D or E, a second reaction was performed using primers for gene C (to differentiate between A and C) or gene E (to differentiate between D and E). The reactions were carried out in an Eppendorf Mastercyler EP gradient 96 well block in a final volume of 25 μL containing 2.5 μl of 10x PCR buffer, 0.4 μl 50 mM MgCl_2_, 1.25 μl dNTP (10 μM) Pool, 2 U Taq DNA polymerase, 0.075 μl (200 μM) of each primer and 2 μl of DNA sample. The conditions for the reactions were as follows: 94°C for 5 min; 30 cycles of 94°C for 30 s, 63°C for 30 s, 68°C for 10 min, and a final extension step of 72°C for 10 min.

### PCR-Based *E. coli* Serogrouping

PCR analysis was used to screen for the most common APEC serogroups, using primers and conditions described by Iguchi et al. (22) with minor modifications in annealing time/temperature to accommodate the melting temperatures of the primers used.

The reactions were carried out in an Eppendorf Mastercyler EP gradient 96 well block in a final volume of 25 μL containing 2.5 μl of 10x PCR buffer, 0.4 μl 50 mM MgCl_2_, 1.25 μl dNTP (10 μM) Pool, 2 U Taq DNA polymerase, 0.075 μl (200 μM) of each primer and 2 μl of DNA sample. The conditions for the reactions were as follows: 94°C for 5 min; 30 cycles of 94°C for 30 s, 58°C for 30 s, 72°C for 2 min, and a final extension step of 72°C for 10 min.

### Pulsed Field Gel Electrophoresis (PFGE)

PFGE was carried out on a select group of isolates from barn A2, the outbreak barn (weeks 17 and 18) from litter, cellulitis lesions and systemic isolates recovered from the organs of birds at necropsy. Isolates were analyzed using the method described by Ribot et al. (23, 24). Preparation, lysis, washing of plugs, and *XbaI* restriction were performed according to the PulseNet protocol using a CHEF mapper XA system (BioRad, Hercules, CA). *Salmonella Braenderup* H9812 was used as the size standard. Macrorestriction patterns generated were compared using the BioNumerics Fingerprinting software (Ver 7.6, Applied Math, Austin, TX). The similarity index was calculated using the Dice coefficient, with a band position tolerance of 1% and an optimization of 0.5%. The unweighted-pair group (UPGMA) method was used to construct the dendrogram as previously described (24).

### Statistical Analysis

Statistical analysis was used to assess the relationship between mortality in the case and control barns compared against the number of samples collected in these barns where APEC-like strains were detected. The non-parametric Wilcoxian-matched-pair signed rank test was applied to allow comparison between data over the total samples and intervals and for individual sampling days i.e., paired data.

For the analysis of virulence and resistance genes harbored by strains examined in the study the number of genes were treated as quantitative variables and the data was analyzed using non-parametric tests also due to asymmetry in the distribution of these genes. Direct comparisons (where possible) between two groups were made using the Mann-Whitney U test. All statistical analysis was performed using GraphPad Prism (Version 7.0d) for MAC OS X (GraphPad, La Jolla, CA). Statistical significance was accepted when *p* < 0.05.

## Results

### Prevalence of APEC Genes and Mortality Rate

**Figure 1:**
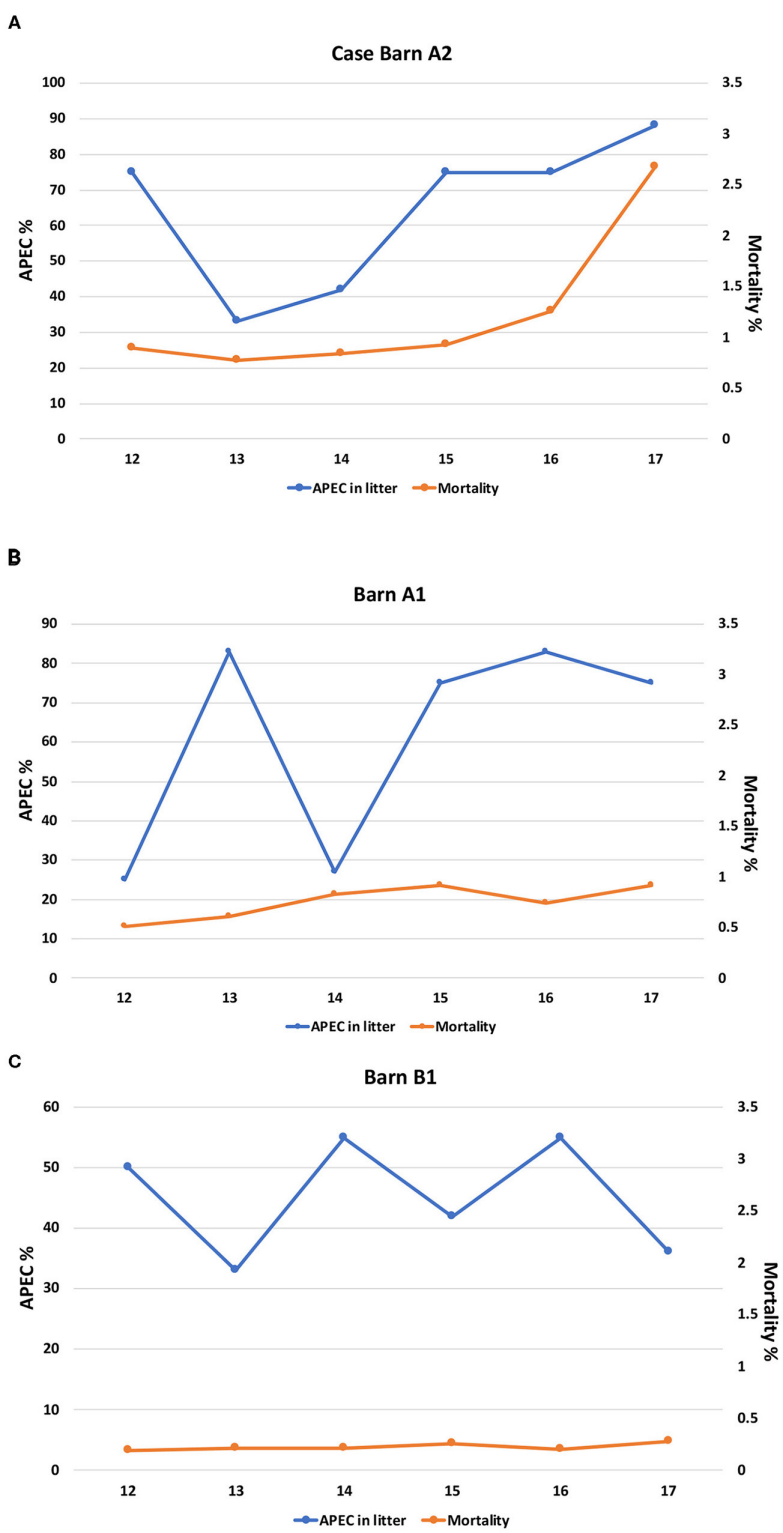
**(A)** Prevalence of APEC-like strains in the litter compared with mortality for the case barn (A2). **(B)** Prevalence of APEC-like strains in the litter and mortality in control barn A1. **(C)** Prevalence of APEC-like strains in the litter and mortality in control barn B1.

To assess whether the quality of the production environment (litter) affected the mortality rate of the birds, we analyzed the relationship between the presence of APEC-like strains (strains harboring at least three of the 5 minimal APEC predictors) in the litter and the mortality rate. In barn A2 flock mortality sharply increased from 1.3 to 2.7% between weeks 16 and 17 which correlated with a spike in APEC-like isolate prevalence in litter samples, which reached 88% at week 17. The prevalence of APEC-like isolates detected in the litter appeared to peak at weeks 14-16 before the mortality rate peaked (Figure 1A). For the two control barns assessed (Figures 1B,C), mortality was relatively constant, hovering around 0.25–0.9 between weeks 12 and 17, with no significant peaks. Highest APEC-like prevalence was >80% at week 16 (Figure 1B). Mortality for barn B1 hovered around 0.25% with APEC-like strain detection of 30–55% among litter samples.

When compared overall, there were no significant differences for APEC detection or mortality for all three barns; in contrast, when compared across the barns (A2 vs. A1) significant differences in APEC prevalence were noted at weeks 12 and 13 only (*p* < 0.05) while comparison of A2 against barn B1 noted significant differences at week 17 only (*p* < 0.05). Similarly, when mortality rates at the case and control barns were compared (A2 vs. A1), significant differences in mortality were noted overall (*p* < 0.05). The same observation was noted when the case barn was compared to the second control barn (A2 vs. B1) with mortality being significantly greater in the case barn (*p* < 0.05) and across all time intervals compared (*p* < 0.05).

### Prevalence of Antimicrobial Resistance Genes

**Figure 2:**
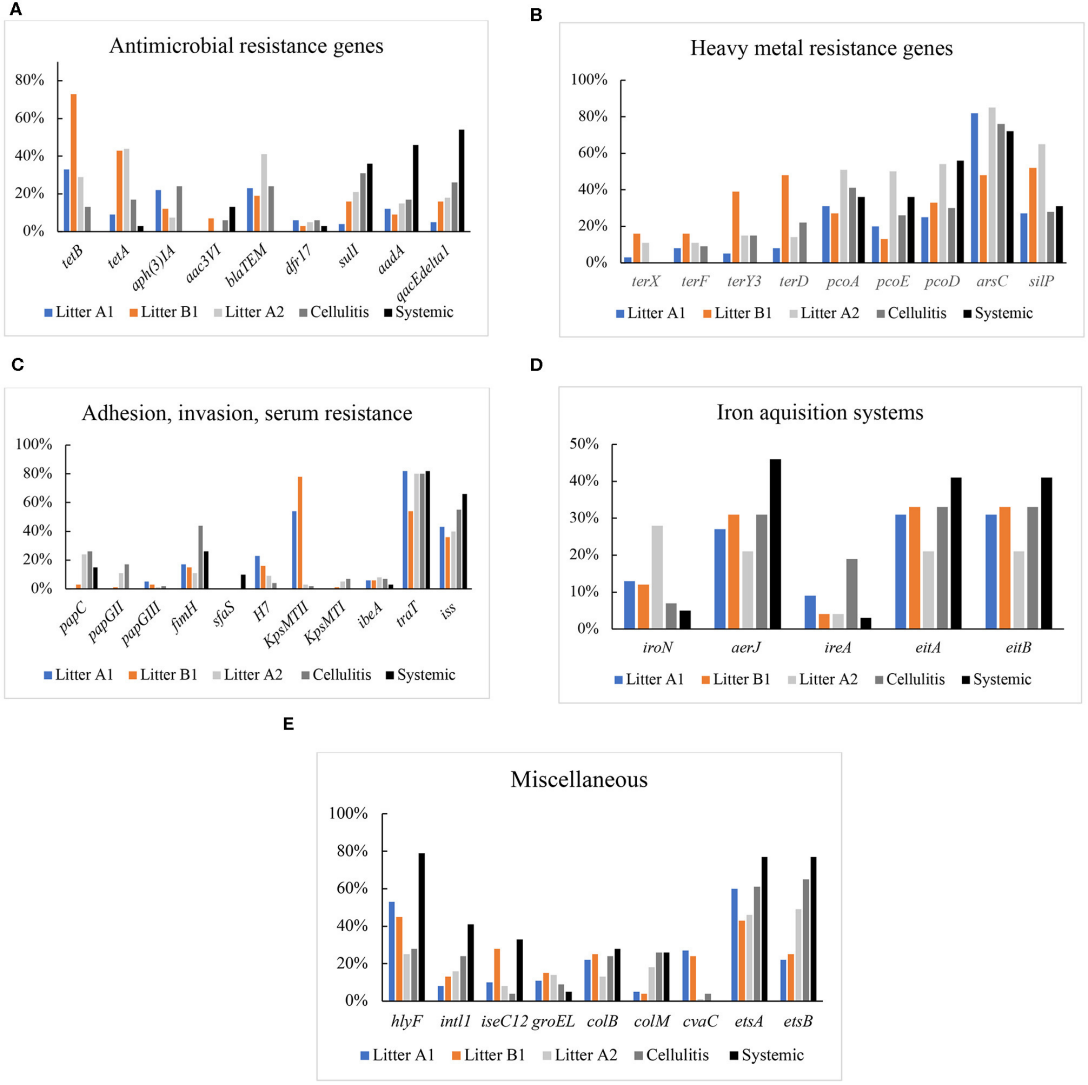
**(A)** Prevalence of genes encoding antimicrobial resistance in Litter, cellulitis, and systemic isolates. **(B)** Prevalence of genes encoding resistance to heavy metal in litter, cellulitis, and systemic isolates. **(C)** Prevalence of genes encoding adhesins, invasins, and serum resistance in litter, cellulitis, and systemic isolates. **(D)** Prevalence of genes encoding iron acquisition proteins in litter, cellulitis, and systemic isolates. **(E)** Prevalence of genes encoding colicins, integrase, transposase, chaperone, and others in litter, cellulitis, and systemic isolates.

Genes encoding resistance to protein synthesis inhibitors tetracycline (*tetA, tetB*), aminoglycosides, gentamicin [*aph(3)IA*] and *aac3VI*, spectinomycin and streptomycin (*aadA*), cell wall synthesis inhibitor ampicillin (*bla*_*TEM*_), DNA synthesis inhibitor trimethoprim (*drf17*), folate synthesis inhibitor sulfonamide (*sulI*), and multidrug resistance (*qacE*Δ*1*) were included in the analysis (Figure 2A). Genes associated with resistance to tetracycline A (*tetA*), gentamicin [*aph(3)IA*], or ampicillin (*bla*_*TEM*_) were not detected in systemic isolates; while these isolates showed high prevalence of genes associated with sulfa (36%), spectinomycin and streptomycin (46%), *qacE*Δ*1* (54%). Among the cellulitis isolates, highest frequencies of resistance genes were found for *aph(3)IA* and *bla*_*TEM*_ (24%), *qacE*Δ*1* (26%), and sulfa (31%). Among litter from control barns (A1 and B1) isolates, high frequencies were noted for *tetA* (43%), *tetB* (33%; 73%), *bla*_*TEM*_ (23%), and *aph(3)IA* (22%). While a high frequency of *tetB* (44%) and *bla*_*TEM*_ (41%) were found in isolates from case barn A2 (Figure 2A).

Isolates were separated out for statistical analysis by source: litter in barn A1 and litter in barn B1 (control barns), and litter in barn A2 (case barn), cellulitis and systemic isolates. When compared by origin, the prevalence of *bla*_*TEM*_ was significantly higher in litter from the case barn (A2) when compared to both A1 and B1 (control barns). The prevalence of *sulI* was significantly higher (*p* < 0.05) in A2 (case barn) than in A1 (control). *tetA* frequency was significantly higher (*p* < 0.05) in A2 than A1, but not when A2 was compared to B1 (second control barn). Surprisingly *tetB* and *aph(3)IA* were significantly higher (*p* < 0.05) in either of the control barns (see Supplementary Table 2).

Comparing cellulitis vs. systemic isolates, *tetA, tetB, aph(3)IA*, and *bla*_*TEM*_ prevalences were significantly higher in cellulitis isolates, while *aadA* and *qacEdelta1* were significantly higher in systemic isolates.

Statistical analysis was also used to compare cellulitis isolates and systemic isolates with isolates recovered from litter of barns A1, B1, and A2. The frequencies of *tetA* and *tetB* were significantly higher (*p* < 0.05) in litter isolates than in both cellulitis and systemic isolates. Similarly, detection of *sulI* was higher in systemic than in control barns litters (A1 and B1) but not in case barn (A2), and in cellulitis isolates when compared to litter A1 (control). The genes *aadA* and *qacEdelta1* were significantly more prevalent in systemic isolates of *E. coli* than in litter from controls (A1 and B1) and case barn (A2).

### Heavy Metal Resistance Genes

Isolates were also screened for the presence of genes that encode resistance to heavy metals, including copper (*pcoA, pcoD, pcoE*), silver (*silP*), tellurite (*terD, terF, terY3, terX*), and arsenicals (*arsC*) (Figure 2B). Genes encoding resistance to tellurite were not detected in systemic isolates; however, a high prevalence of *arsC* (72–76%), *pcoAD* (26–56%) and *silP* (28–31%) were detected in both systemic and cellulitis isolates. In Litter A2 isolates, the prevalence of *arsC* (85%)*, pcoAD* (50–54%), and *silP* (62%) was higher than those in Litter A1 and Litter B1.

When compared by origin, detection of *pcoADE* was significantly higher in case barn A2 than in both control barns (A1 and B1). *arsC* frequency was significantly higher in A2 than A1 but not B1. *terY* and *terD* frequencies were unexpectedly higher in litter B1 (control) than A2 (case).

When cellulitis and systemic isolates were compared, detection of *terY* and *terD* was significantly higher in cellulitis isolates, while *pcoD* was higher in systemic isolates.

We also compared cellulitis and systemic isolates with isolates from the litter of barns A1, B1, and A2 finding *pcoD* prevalence was higher in systemic isolates when compared to control barns but not to the case barn.

### Adhesins, Invasins, and Protectins

The prevalence of genes encoding adhesins (*papC, papEF, papGII, papGIII, fimH, sfaS, h7, bmaE*), invasins (*ibeA*), and protectins, involved in the interaction of APEC with host cells and in survival in host serum (*kpsMTI* and *kpsMT2, traT, iss*) was also analyzed (Figure 2C).

Among systemic isolates, the adhesion genes *papG* (II and III), *kpsMT* (I and II), and *h7* were not detected*. sfa, papC*, and *fimH* were detected 10, 15, and 26% of systemic isolates, respectively. The invasion *ibeA* was found in only 3% of isolates examined. A high prevalence of serum resistance *iss* (66%) and *traT* (82%) was found. With regards to fimbrial subunits, H7 and *sfaS* were either not detected or detected at a very low prevalence (4–10%). In contrast, *fimH* (the adhesive subunit of Type 1 fimbriae) showed a high prevalence, in litter (63%) and cellulitis (44%) isolates. The overall frequency of genes of the operon *pap* (pyelonephritis-associated pilus) was low, with *papC* being the most frequent and present in only 15% of systemic isolates, 21% of litter isolates and 26% of cellulitis isolates. The invasion gene *ibeA* was found in 2% of systemic isolates, and 6–8% of cellulitis and litter isolates. P adhesin alleles were only found in litter isolates with a frequency of 3%. K1 capsule was absent in systemic isolates, and present in only 2% of litter isolates and 7% of cellulitis isolates. Heme-agglutinin *bmaE* was found in <1% of litter isolates. High frequencies were found for serum resistance genes *traT* (80–82%) and *iss* (40–66%). Genes encoding proteins involved in complement resistance (*traT*) and serum survival (*iss*) were also analyzed. *traT* was detected in 82% of systemic isolates, 80% of cellulitis isolates and 73% of litter isolates. *iss* prevalence was high in all three groups of isolates analyzed: 66% of the systemic, 55% of the cellulitis, and 40% of the litter isolates harbored the gene.

When analyzing litter isolates by origin, *traT* was significantly higher (*p* < 0.05) in A2 than in B1 but not in A1. Surprisingly, *papG2/3* and *fimH* were significantly (*p* < 0.05) more prevalent in litter B1 (control barn) than in A2 (case barn).

When cellulitis vs. systemic isolates were compared, the frequency of *papGII* was significantly (*p* < 0.05) higher in cellulitis isolates, while *sfaS* was significantly higher in systemic isolates.

In a comparison between cellulitis and systemic isolates with isolates from litter of barns A1, B1, and A2, *fimH* was higher in litter from all three barns when compared to systemic isolates. *traT* was higher in cellulitis and systemic isolates than in litter from barn B1 (but not A1 and A2). *iss* was significantly higher in systemic than in litter isolates.

### Iron Acquisition Systems

The iron-scavenge related genes analyzed in this work included *fyuA, iroNEC* (salmochelin), *ireA* the iron-regulated outer membrane protein, aerobactin (*aerJ), eitA*, and *eitB* (ABC iron-transport system) (Figure 2D). *fyuA* was not detected in any isolate. The most prevalent iron-related genes in systemic, cellulitis, litter A1, and litter B1 isolates were *aerJ* (27–46%), and *eitA* (31–41%), and *eitB* (31–41%). In litter A2 isolates, *iroN* (28%) had the higher prevalence, followed by *aerJ, eitA*, and *eitB* (21%) (Figure 2D).

When litter isolates were analyzed by source, the prevalence of *iroN* was significantly higher in litter A2 (case) than in A1 and B1 (controls). No significant differences were observed between the frequency of the other iron acquisition genes analyzed among litter from different origins.

With regards to cellulitis vs. systemic isolates, *ireA* was found to be significantly higher in cellulitis isolates. No other significant differences were observed in iron acquisition genes between cellulitis and systemic isolates.

In a comparison between cellulitis and systemic isolates and litter isolates of A1, B1, and A2, *aerJ* was more prevalent in cellulitis isolates than in litter isolates from case (A2) and control barn (A1). Frequencies of *eitA* and *eitB* were higher in systemic than in litter isolates from case barn (A2).

### Miscellaneous

Several other virulence genes were also analyzed in this study (Figure 2E). *hlyF* (hemolysin) showed a much higher prevalence (79%) in systemic isolates when compared to cellulitis (27%) and litter (41%). Genes encoding colicins were also analyzed. *cvaC* (structural gene for colicin V) was absent in systemic isolates but detected in 4% of the cellulitis isolates and 18% of the litter-associated isolates. *colB* (colicin B) was detected in 26% of the systemic and cellulitis isolates, 9% of the litter-associated isolates, and *colM* (colicin M) was present in 28% of the systemic, 24% of the cellulitis and 20% of the litter isolates. The presence of the genes encoding chaperone GroEL, integrase IntL, and the transposase IseC12 was also analyzed (Figure 2E).

Additional virulence factors *malX, papA, cnf* (cytotoxic necrotizing factor), *fyuA* (siderophore), *sfa* (S fimbriae), *hlyD* (alpha-hemolysin transport), *rfc* (replication factor C), *papGI, gafD* (fimbrial gene cluster), *cdtB* (cytolethal distending toxin), *focG* (F1C minor fimbrial subunit), *iha* (enterobactin receptor/adhesion), and *afa* (afimbrial adhesion) were not found in any of the isolates examined.

When litter isolates were compared by origin, the only gene significantly higher in case vs. control barns was *colM*. In contrast, *cvaC* and *hlyF* was significantly lower in case barn than in both controls.

With regards to cellulitis vs. systemic isolates, *hlyF* and ISEc12 were more prevalent in cellulitis than in systemic isolates. No other differences were found between frequencies of miscellaneous genes between cellulitis and systemic isolates.

Comparing miscellaneous genes between systemic and cellulitis isolates and those of litter origin, we found that *hlyF*, and *intI* were more frequent in systemic than in litter isolates from all barns; *ISEc12* was more prevalent in systemic than in litter from barns A1 and A2. *colM* was higher in systemic and cellulitis isolates when compared to litter isolates from control barns. *etsA* and *etsB* were higher in systemic than in litter from case (A2) and control (B1) barns.

### Phylogenetic Groups

PCR-based phylogenetic typing was performed according to the method described by Clermont et al. (18), which assigns *E. coli* strains to groups A, B1, B2, C, D, E, and F, according to the presence of *chuA, yjaA* and *arpA*, and the DNA fragment *TSPE4.C2*. Most systemic isolates classified as B2 (31%), A (28%), and C (23%), with the remaining isolates classified as F (15%) and D (3%). The majority of cellulitis isolates were classified as phylogenetic groups A (41%), F (25%), and B2 (22%), with the remaining isolates belonging to phylogenetic groups B1 (8%), and C (4%). Litter isolates were primarily assigned to group A (41%), followed by B1 (33%), with the remaining isolates classified as B2 (10%), D (6%), and F (6%) (Table 2). Litter isolates from barn A2 were primarily classified as A (48%), B1 (18%), and B2 (17%) (Table 2). Litter samples were divided in two groups based on the presence of the APEC minimal predictors: “APEC-like” (3 or more of the minimal APEC predictors) or non APEC-like (2 or less of the minimal APEC predictors). Regardless of the presence of APEC predictors, most of the isolates were assigned to phylogenetic groups A and B (data not shown).

**Table 2:** Phylogenetic classification of isolates examined.

	**Litter A1**		**Litter A2**		**Litter B1**		**Cellulitis (*****n*** **= 54)**		**Systemic (*****n*** **= 39)**	
**Group**	***n***	**%**	***n***	**%**	***n***	**%**	***N***	**%**	***n***	**%**
A	39	42	37	48	19	28	22	41	11	28
B1	27	29	14	18	37	55	4	7	0	0
B2	6	7	13	17	4	6	12	22	12	31
C	4	4	3	4	2	3	2	4	9	23
D	10	11	4	5	1	1	0	0	1	3
E	0	0	1	1	0	0	0	0	0	0
F	6	7	5	6	4	6	13	24	6	15

### PCR-Based Serogrouping

**Figure 3:**
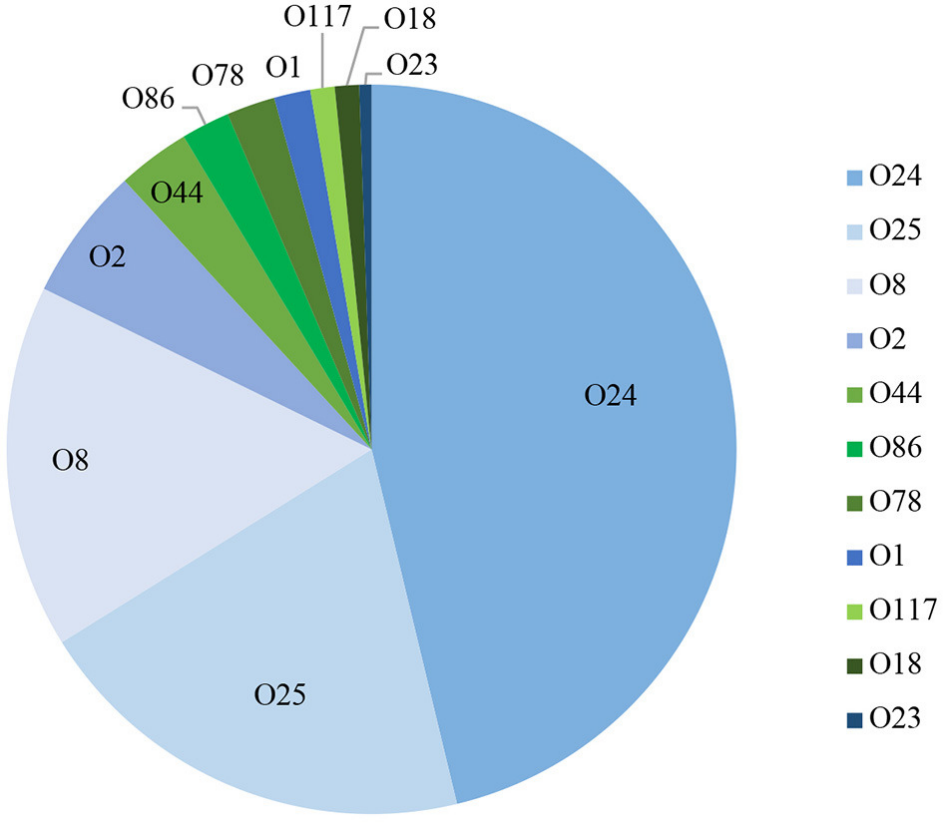
PCR based O-typing. Distribution of APEC most common O-types among litter, cellulitis and systemic isolates includes only isolates that were assigned to one of the O-types screened.

Using a PCR-based method we were able to determine the O-group of 186 isolates. In summary, the most prevalent serotype among the typed isolates was O24 (46%), followed by O25 (20%) and O8 (16%) (Figure 3). When examined by source, 60% (144) of the litter isolates were typeable, 57% (25) of the cellulitis isolates and 28% (11) of the systemic isolates. O-types O24, O25 and O8 were the most frequently detected serogroup in all three groups of isolates (Table 3 and Figure 3).

**Table 3:** O-type distribution according to origin of isolates.

	**Litter A1**		**Litter A2**		**Litter B1**		**Cellulitis**		**Systemic**	
	***N***	**%**	***N***	**%**	***n***	**%**	***n***	**%**	***n***	**%**
O1	0	0	2	4	0	0	0	0	1	9
O2	3	5	2	4	3	7	2	6	1	9
O8	12	22	1	2	14	34	2	6	2	18
O17	0	0	0	0	0	0	0	0	0	0
O18	0	0	2	4	0	0	0	0	0	0
O23	0	0	0	0	0	0	0	0	1	9
O24	33	60	18	38	15	37	17	55	3	27
O25	5	9	21	44	3	7	5	16	3	27
O44	0	0	0	0	2	5	4	13	0	0
O78	1	2	2	4	0	0	0	0	0	0
O86	0	0	0	0	4	10	0	0	0	0
O117	1	2	0	0	0	0	1	3	0	0

### PFGE Analysis

**Figure 4:**
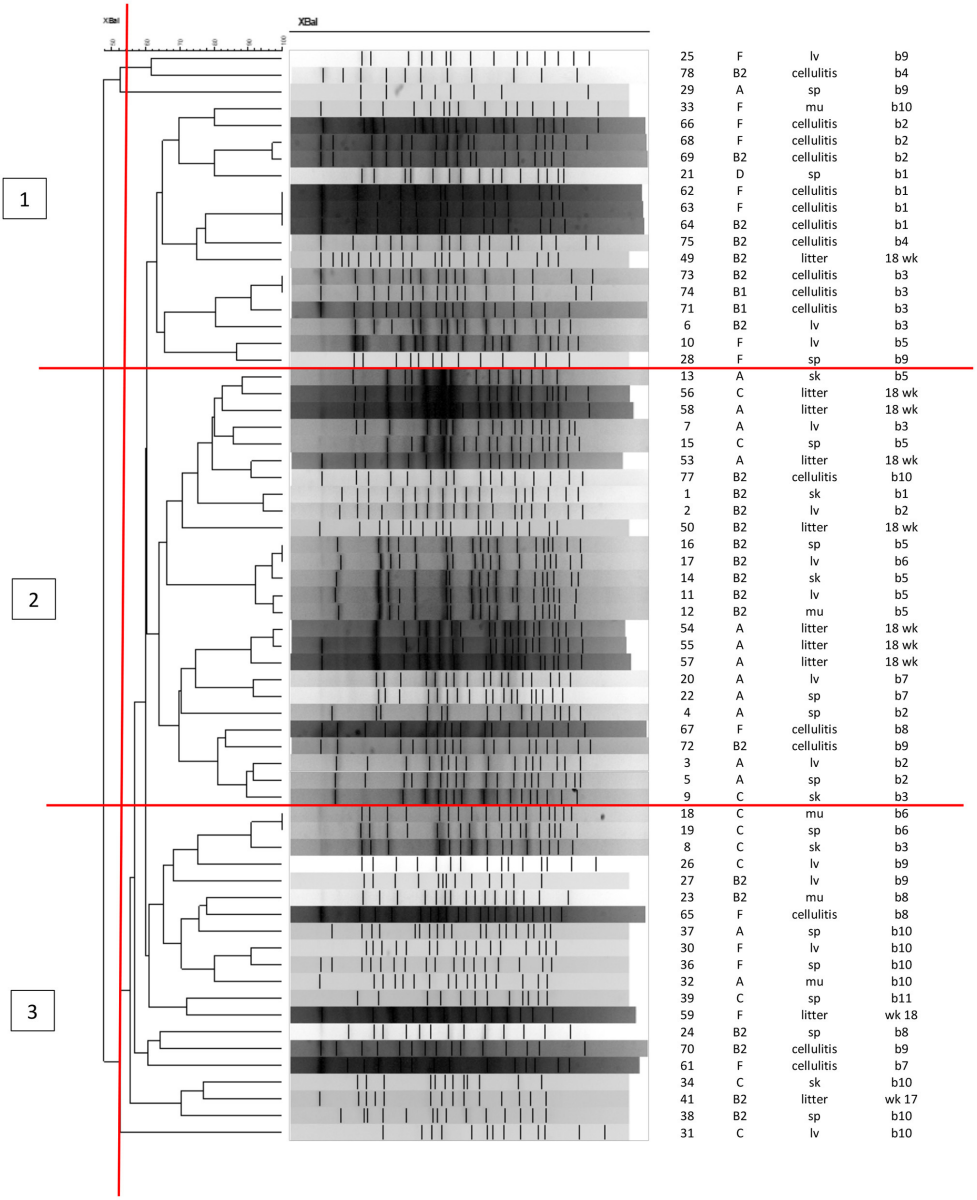
PFGE analysis of APEC and APEC-like strains analyzed at 17 and 18 weeks in litter, cellulitis and systemic strains from barn A2. First column shows the isolate ID, column 2 shows the phylogenetic group, column 3 the source of isolate (litter, cellulitis, lv, liver; sp, spleen; sk, skin; mu, muscle), column 4 is the bird identification numbered 1–11 and week litter was collected (wks 17 or 18).

Figure 4 shows the PFGE profiles and dendrogram generated for 65 *E. coli* isolates: 38 were recovered from the skin and organs of 11 birds at necropsy (indicated by tissue of isolation); 17 isolates recovered from cellulitis lesions and 10 litter isolates collected from the four quadrants in barn A2 at weeks 17 and 18 of age. About 10 other isolates from litter failed to restrict using *XBaI*. At about 55% identity, the data classified PFGE patterns generated into three major clusters with overlaps in the strains found in each cluster. Cluster 1 was dominated by cellulitis strains and also included some systemic strains as indicated by organ of isolation; cluster 2 consisted primarily of litter and systemic strains while cluster 3 consisted primarily of systemic strains and some cellulitis strains. Of note, none of the exact same PFGE patterns were detected across all three sources, however there was a similar pattern detected from two individual birds (bird 6 and 7) where the same strain was recovered from a spleen in one and a liver in the second (isolate numbers 16 and 17). Similar patterns were also noted among cellulitis swabs taken from the same bird but internal cultures from tissues of these birds did not match suggesting that the infection was not exclusive to a single strain and likely was impacted by more than one disease causing strain. Among litter isolates from the same time-frame no matches with the disease strains were found but isolates in cluster 2 showed some clustering of similar patterns that were likely related and consisted of both the disease strains and litter but the PFGE restriction patterns were not identical.

## Discussion

Data on modeling the prevalence of APEC in turkey barns and concurrent outbreaks and mortality associated with cellulitis is relatively limited and this study may be one of the first to use such approach in analysis (Figures 1A,B). Of significant importance is the increased prevalence of APEC-like strains in the outbreak barn (Figure 1A) prior to increased mortality i.e., the pathogen prevalence peaked before the disease. While multiple factors contribute to increased mortality in production birds, including the presence of other pathogens, parasites, the immune status of the birds, and other stressors both physical in the bird and the environment, this study does provide evidence of a peak in the prevalence of APEC-like isolates in the litter prior to the mortality peak suggesting that APEC may have played a role in contributing to the disease.

One of the limits of the current study however is that our analysis is based on an outbreak in one barn (A2) with a previous history of cellulitis and despite our best efforts it was not possible to identify any additional barns at the time of the study either on this farm or in the locale. This study does, however, provide novel insight into *E. coli* (APEC) associated with the outbreak and its potential role in the disease and the use of litter as an indicator of the health status of the barn. Further studies are needed to better understand APEC's role in cellulitis, however as this study is based on a naturally occurring outbreak—it is not always possible to predict where and when cases will occur, but where there is a history of outbreaks, continued monitoring is likely warranted.

In this study, the occurrence of 64 virulence-associated genes in 333 *E. coli* isolates obtained from litter, cellulitis and systemic lesions from turkeys from two different farms in Iowa were analyzed (Figures 2A–E). Gene screening included genes associated with antimicrobial resistance, heavy metal resistance, iron acquisition systems, adhesion and invasion proteins and a collection of miscellaneous genes including transposase, integrase, chaperones, and toxins.

This study provides a unique comparison of and likely is one on the largest reports assessing virulence genotyping of *E. coli* strains associated with turkey production with a particular focus on a barn associated with a cellulitis outbreak.

The highest frequencies of antimicrobial resistance genes were found in systemic isolates and included the multidrug resistance marker (*qacE*Δ*1*), streptomycin (*aadA*), and sulfonamides (*sulI*). High levels of sulfonamide resistance genes found is in agreement with previous studies of pathogenic and commensal *E. coli* of turkey origin in Italy (26). A high frequency found for the streptomycin resistance (*aadA*) gene in systemic isolates also agrees with a similar study found in a collection of *E. coli* of turkey origin from Italy (27). It has been reported that resistance to streptomycin might be prevalent despite the discontinuance of its use (28). Resistance associated with ampicillin (*bla*_*TEM*_) and gentamicin (*aph(3)IA*) were not found in systemic isolates examined in the current study.

Among the litter isolates examined, highest frequencies of resistance gene presence were noted for *bla*_*TEM*_ (28%), tetracyclines A (30%), and B (43%). High resistance against tetracycline in pathogenic and commensal *E. coli* from turkey have been reported (27).

Our collection was also screened for the presence of *intI1, dfrA1* and *aadA1*. *intI1* encodes a class 1 integron that are known to play a role in acquisition and dissemination of antimicrobial resistance (29), and class 1 integrons are the most frequently detected and the best characterized, especially in *Enterobacteriaceae* (30). Previous studies have shown an association between the presence of class 1 integrons and the *aadA1* and *dfrA* resistance cassettes (27). Here, we found that 34 (58%) out of 58 isolates that harbored *intI1* gene were also positive for *aadA*, which provides additional evidence for the relationship between class 1 integrons and resistance to aminoglycosides.

Previous work on virulence genotyping in *E. coli* from turkey have focused on genes that are significantly associated with highly pathogenic APEC strains, including *iutA, hlyF, iss, iroN* and *ompT, iucD, fyuA, irp-2, tsh, fimC* and *papC, sit A, cvi/cva* (26, 31–33). Consequently, there is limited information on the frequency of most of the genes analyzed in this study in *E. coli* of turkey origin as a whole and this paper provides some new insight into virulence associated with cellulitis *E. coli* of turkeys.

The prevalence of three of the five genes determined as minimal predictors of APEC: the increased serum survival *iss*, hemolysin *hlyF* and the ferric aerobactin receptor *iutA* found in this work is comparable to the similar reports from previous studies. *iss* frequencies found were 40% in litter, 55% in cellulitis, and 66% in systemic isolates. This data agrees with previous work analyzing isolates from turkeys with colibacillosis from Brazil, where *iss* was detected in 93% and 64% of their isolates, respectively (31, 32). Another study analyzing *E. coli* from cecal swabs of healthy turkeys found *iss* in 55% of the isolates (33). *hlyF* was detected in 79% of the systemic isolates analyzed in the current study, compared to 81% found in systemic isolates in Brazil (32). *iutA* was detected in 46% of our systemic isolates compared to 64% in previous work (32).

The prevalence of the other two genes of the APEC pentaplex (12), the outer membrane protein *ompT* and the membrane siderophore receptor *iroN*, was surprisingly low in the systemic isolates analyzed in this work. *ompT* was found in only 8% compared to 81% from previous work (32). *iroN* was found in only 5% of systemic isolates in this work, but have been previously found in 69% (32) and 95% (31) of isolates examined elsewhere. Reasons for these differences are currently unknown and will warrant further investigation.

Other genes analyzed in this and previous works include *fimH, K1, papC, ibeA, cvaC*, and *fyuA. fimH* was detected in 63% of litter isolates in this work. Similarly, high frequency of *fimH* (97%) was reported in a collection of *E. coli* from cecal swabs from healthy turkeys in the UK (33). The frequency of *fimH* in litter and cellulitis isolates in this study were 63 and 44%, respectively. The capsule *K1* gene was detected in only 2% of litter and 7% of cellulitis isolates, which is in agreement with the low prevalence (4%) found in healthy turkey isolates elsewhere (33). The prevalence of *papC* was 21% in litter and 26% in cellulitis isolates. In systemic isolates the prevalence of *papC* was 15%, which was relatively similar to systemic isolates from turkeys with airsacculitis in Brazil (31). In contrast, the presence of *papC* in fecal isolates from healthy turkeys in UK was only 3% (33).

*cvaC* was not detected in any systemic isolate tested in the current study. This was unexpected, as previous studies have detected *cvaC* in 67 and 34% of systemic isolates examined (31, 32). In litter isolates, *cvaC* frequency was 18%, comparable to what was found in fecal isolates from healthy turkeys in the UK (33). *fyuA*, found in 45% of systemic isolates in a study from Brazil (32) was detected in only 1 systemic isolate in the current study. The invasion *ibeA* previously found in 31% of systemic isolates (31) in Brazil, was found in only in 3% of systemic isolates, and 7% of cellulitis and litter isolates in this study.

A surprising number of *E. coli* from all three sources examined could be serogrouped and the most common serogroups implicated included O:24, O:25 and O:8 and O:2 with smaller prevalence associated with serogroups O44, O:86, O:78, O:1, O:117, O:18, and O23. Among APEC implicated in disease O:1, O:2, and O:78 are most often associated with disease of poultry including turkey (13, 26, 34) and often represent the majority of serogroups present. These serogroups have been reported in outbreaks dating back to the 1960s (25, 35, 36) however, in the current study they represented a small fraction of the isolates examined. More recently, however, newer serogroups including O18 and O111 have been found in *E. coli* isolated from turkeys with hemorrhagic septicemia (37) suggesting there are changes in the dominant serogroups causing disease. Of significant interest however was the high prevalence of O:24 and O:25 serogroups among isolates implicated in disease (both cellulitis lesions and on systemically infected organs) and in the litter. These would appear to be new serogroups that are not frequently implicated in disease and may therefore be emergent. Current data on *E. coli* serogroups implicated in disease of turkeys is, however, relatively limited thus curtailing our ability to perform adequate comparative analysis. Regardless, careful monitoring for changes in serogroups causing disease is warranted as there may be factors responsible for shifts in the serogroups and the selection of new types that are currently unknown.

The phylogenetic analysis of litter isolates found they primarily classified as phylogenetic group A (40%) followed by B1 (33%). Cellulitis isolates were mostly A (41%) followed by F (24%) and B2 (22%), whereas systemic isolates where mostly B2 (31%) followed by A (28%) and C (23%). In previous work on *E. coli* isolated from turkeys with airsacculitis in which the earlier Clermont phylogenetic typing scheme (19) was used that classified strains as A, B1, B2, and D, it was found that 50% of the isolates belonged to group B2, 28% to group B1, 17% to group A and 5% to group D (31).

When source of strains were compared overall it was found that there were different classification of the isolates when source types were compared. For example, almost 80% of the litter isolates were represented by phylogenetic groups A, B1, and F, in each of the barns and this prevalence was similar for cellulitis isolates (90%) and but dropped for systemic isolates to 43%. In contrast, the B2 phylogenetic group was represented in 10–20% of the litter isolates from each barn but was significantly greater among strains from cellulitis (22%) or systemic disease (31%). Assignment to phylogenetic group B2 is considered a trait of ExPEC (18, 19). Isolates of phylogenetic group C represented 23% of the systemic isolates but among all other groups was <5% prevalence. This data does however continue observations of earlier studies of this lab who noted that APEC were represented in the other phylogenetic groups aside from B2 to a greater extent (38), suggesting that APEC do not classify as easily by the Clermont method (18). This data does however also highlight the opportunistic nature of some of these APEC and APEC-like strains especially those of litter toward disease and likely is linked to additional virulence traits carried by these organisms, and litter should not be excluded as a potential source of pathogenic organisms.

PFGE Analysis of strains of APEC and APEC-like strains from the litter, lesions and tissues associated with cellulitis in the outbreak barn (A2) at its peak—i.e., weeks 17 and 18 failed to identify identical patterns across all three sources for strains implicated in disease. This observation was not however unusual as our research group has found this before and its more likely we do not find matching patterns due to the diverse nature of *E. coli* found in the environment and in disease (24, 39). In addition, the sample collection for this analysis is relatively small which may also limit our findings. Of interest however, were identical patterns found in two different birds both isolated from organs suggesting that there may be some circulating clones causing disease.

### Overall Conclusion

Turkey cellulitis continues to be a top health concern in the turkey industry, and despite *Clostridium septicum* being implicated as a major pathogen, a number of opportunistic bacteria including *E. coli* have been implicated. Isolates of *E. coli* (APEC) recovered from cellulitis lesions and systemic infection such as those examined in this study are well-developed pathogens and should not be overlooked when exploring causes of cellulitis in turkeys. Of interest would be to assess the impact and role of APEC in the disease process, which will warrant further research. As the poultry industry trends toward restricted antibiotic use, prevention and control strategies has become increasingly important. Regardless, the role of pathogenic *E. coli* in the disease process should not be overlooked.

## Data Availability Statement

All datasets generated in this study are included in the article/Supplementary Material.

## Ethics Statement

The work presented was covered under the Institutional Biosafety Committee (IBC) approval at the University of Georgia and under IBC and IACUC at Iowa State University. Written informed consent for participation was not obtained from the owners because verbal consent was part of a veterinarian visit to assess birds.

## Author Contributions

AO carried out the research, data analysis, and drafting of the paper. DN performed analysis for genes and co-authored the paper. YS provided assistance in sampling necropsies, analysis of farms and farm visits, and contributed to writing the paper. AN contributed to sampling at the farms and at necropsy, contributed to drafting the paper, and microbial analysis. BR contributed to sampling at the farms and at necropsy. LN provided assistance in drafting the paper and provided supplies for the study. NB provided assistance in testing strains and drafting the paper. CL helped design the study, draft the paper, and provided materials for the study. All authors contributed to the article and approved the submitted version.

## Conflict of Interest

The authors declare that the research was conducted in the absence of any commercial or financial relationships that could be construed as a potential conflict of interest.

## Supplementary Material

Supplementary Table 1 Data on isolates used in this study.

Supplementary Table 2 Statistical analysis tables for isolates analyzed in this study.
